# A retrospective clinical study of fixed tooth- and implant-supported prostheses in titanium and cobalt-chromium-ceramic: 5–9-year follow-up

**DOI:** 10.1007/s00784-022-04559-x

**Published:** 2022-06-29

**Authors:** Sheida Nilsson, Victoria Franke Stenport, Marco Nilsson, Catharina Göthberg

**Affiliations:** 1 The Sahlgrenska Academy, Department of Prosthodontics, Institute of Odontology, University of Gothenburg, Box 450, 405 30 Göteborg, Sweden; 2 School of Education and Communication, Jönköping University, Box 1026, 551 11 Jönköping, Sweden; 3 The Institute for Postgraduate Dental Education in Jönköping, Box 1030, 551 11 Jönköping, Sweden

**Keywords:** Dental materials, Cobalt-chromium, Titanium, Fixed prosthodontics, Fixed tooth–supported protheses, Fixed implant–supported protheses

## Abstract

**Objectives:**

The aim of this retrospective study was to evaluate the clinical outcome of fixed tooth- and implant-supported protheses manufactured in porcelain veneered cobalt-chromium (CoCr) or titanium with a follow-up period of 5–9 years.

**Materials and methods:**

This study included 63 patients with a total of 86 fixed dental protheses (FDPs) (53 implant-supported and 33 tooth-supported). In total, 67 were short-span FDPs (3–5 units) and 19 were long-span FDPs (6–12 units). The FDPs were evaluated using a modified version of the California Dental Association (CDA).

**Results:**

The binary regression analysis indicated that neither CoCr nor titanium had a statistically significant effect on the odds of success or survival of either tooth- or implant-supported FDPs. However, the success of FDPs was negatively affected by greater FDP *length* and *general tooth wear*. The survival of FDPs was negatively affected by increased FDP *longevity*.

**Conclusions:**

This study found no statistically significant effect on the odds of success and survival outcomes for any combination of tooth-supported, implant-supported, porcelain-veneered CoCr, or porcelain-veneered titanium FDPs. As the number of FDPs was limited, the results should be interpreted with caution.

**Clinical relevance:**

This study shows that the choice between CoCr porcelain and titanium porcelain in fixed protheses did not have a statistically significant effect on the outcome.

## Introduction

Over the last few decades, the choice of materials for dental restorations has undergone major changes. All ceramic reconstructions are increasingly used in both tooth- and implant-supported prostheses. However, many dentists still prefer metal-ceramic reconstructions [1]. There are many dental alloys on the market [2], and the choice of alloy can sometimes be difficult for the dentist. Several factors determine the choice of material in dentistry, i.e., the rigidity of the alloy, biocompatibility, risk of porcelain fractures, and cost.

As gold alloy has the longest history of use in metal-ceramic constructions, most long-term studies investigate gold alloy [3–9]. A survey conducted among prosthodontists showed that the most used material for tooth-supported short- and long-span fixed dental prostheses (FDPs) is porcelain-veneered cobalt-chromium (CoCr) [1]. The same study also reported that the most used materials in implant-supported partial FDPs are porcelain-veneered titanium and porcelain-veneered CoCr. In the edentulous jaws, implant-supported FDPs are predominantly made of titanium-acrylic [1]. Moreover, a survey conducted among dental laboratories indicated that the most common dental material in fixed prosthodontics is CoCr [10].

The oxidation on the CoCr surfaces creates high corrosion resistance but makes the porcelain firing more technique-sensitive compared to gold alloy. The bond between porcelain and gold has been considered superior compared to the bond between porcelain and CoCr. However, the difference in porcelain bond strength has almost been evened out due to alterations in CoCr alloy composition. Therefore, similar high-fusing porcelains can be used for both gold and CoCr alloys [11].

In addition to high corrosion resistance, CoCr alloys have a higher e-modulus compared to gold alloys and titanium. The high e-modulus is a decisive mechanical property in long-span, cantilever FDPs, and in cases where the vertical space for the prosthetic material is limited. The higher the e-modulus, the higher the resistance to bending of the metal framework, which reduces the risk of porcelain fracture [12].

Unlike CoCr, titanium (commercially pure titanium) has a stiffness, which is within the range of gold alloys. As titanium undergoes a structural change at 882.5 °C, low-fusing porcelain is required, which can lead to porcelain fractures [13]. Therefore, some dentists avoid using titanium-porcelain in dental constructions. When the temperature reaches or exceeds 882.5 °C, the oxide layer on the titanium surface increases, which results in inferior porcelain bonding [14]. As firing porcelain on titanium is technique-sensitive, a very precise oven temperature is required to achieve a good bond [15].

Despite the frequent use of CoCr and titanium, few long-term follow-up studies have compared the survival or success of tooth- and implant-supported porcelain-veneered CoCr and titanium FDPs. A systematic review of all-ceramic and metal-ceramic tooth-supported FDPs with a follow-up of at least 3 years included 15 studies on metal-ceramic FDPs. However, only three of the studies evaluated porcelain-veneered CoCr-porcelain, only one evaluated porcelain-veneered titanium, and the rest evaluated gold-porcelain FDPs. The estimated survival rate of metal-ceramic FDPs, based on all three of the above-mentioned alloys, was 94.4% after 5 years. For porcelain-veneered CoCr and titanium, the estimated 5-year survival rates were 93.3–100% and 94.2% respectively. The cumulative 5-year complication rate regarding framework fracture, fracture of the ceramic, ceramic chipping, and loss of retention for metal-ceramic FDPs (gold, CoCr, and titanium) was 16.3% [16].

In a retrospective study from 2013, included in the systematic review above, 122 patients who received porcelain veneered CoCr FDPs were examined. The cumulative success rate after 5 years was 88.3%, and the survival rate was 92.8% [17].

Another systematic review compared the survival and complication rates of tooth- and implant-supported FDPs. The survival rate for metal-ceramic tooth-supported FDPs was 89.1% after 10 years, and the survival rate of implant-supported metal-ceramic FDPs was 96.7% after 5 years. The technical complications with implant-supported FDPs were significantly higher compared to tooth-supported FDPs. The most frequent technical complications were veneer fractures, abutment or screw loosening, and loss of retention [18].

Due to the increasingly frequent use of CoCr and titanium in dentistry, more long-term follow-up studies are needed. To the authors’ best knowledge, no previous clinical studies have compared porcelain-veneered CoCr and titanium in tooth- and implant-supported FDPs.

The aim of the present retrospective study was to evaluate the clinical outcome of tooth- and implant-supported FDPs manufactured in porcelain-veneered CoCr or titanium with a follow-up period of 5–9 years. The first hypothesis was that there are more technical complications with porcelain-veneered titanium constructions than porcelain-veneered CoCr constructions. The second hypothesis was that implant-supported FDPs have an increased technical complication rate compared to tooth-supported FDPs.

## Material and methods

### Study population

This retrospective cohort study includes patients from two specialist clinics in Sweden. The study population included 113 patients who had received partial tooth- and/or implant-supported FDPs in porcelain -veneered CoCr or titanium delivered by three prosthodontists between January 2007 and December 2010. Patients suitable for the study were identified through a program (T4 RapportGenerator) within the medical record system that was used in both clinics. The exclusion criteria were FDPs smaller than three units and with a shorter function time than 5 years. The identified patients were invited to a follow-up examination. All patients were sent a letter about the study and contacted by phone to make an appointment. All participants signed an informed consent form.

### The clinical examination

The clinical examinations were performed by one dentist who was not responsible for the original treatments. The clinical examinations were performed between May 2016 and April 2017. Information about previous technical complications and repairs was collected from patient records. The clinical examinations were performed blindly regarding the FDP material.

The clinical examination of all patients was performed according to the same protocol, which started with a short interview. The interview included questions about whether the patients were satisfied with the reconstruction, including aesthetic and functional opinions.

After the interview, the FDPs were evaluated clinically. Surface integrity was graded using a modified version of the California Dental Association (CDA) evaluation system (Table 1). The quality system (CDA) has two categories: *satisfactory* and *not acceptable*. *Satisfactory* consists of two sub-ratings: R (Romeo) and S (Sierra). R is defined as excellent and S as acceptable. Not acceptable consists of sub-ratings T (Tango) and V (Victor), which are defined as retrievable and unacceptable, respectively. The final assessment of each restoration is based on the lowest sub-rating. If the information in the journal indicated that an FDP had been repaired or remade at a laboratory, the outcome was registered as V. A restoration was regarded as a *success* if all the registrations were Romeo (excellent) or Sierra (acceptable). A restoration with R, S, and T registrations was regarded as *survival*, and V was regarded as *failure*.

**Table 1:** Surface according to the modified California Dental Association protocol

Score	Criteria
Romeo (excellent)	The surface of the restoration is intact
Sierra (acceptable)	The surface of the restoration is slightly rough or pitted and can be polished but is unnecessary
Tango (retrievable)	Superficial fracture, no effects on the function, can be polished
Victor (unacceptable)	Fracture affecting function cannot be corrected by polishing (e.g., loss of occlusal contacts)

In addition to the assessment of the FDP quality (e.g., fractured porcelain or framework, and loss of retention), occlusion, articulation, and general tooth wear (attrition) were registered.

### Statistical analysis

A chi-squared test was used to assess the level of patient satisfaction with regard to the FDPs of the two materials. The success and survival of tooth- and implant-supported FDPs in porcelain-veneered CoCr or titanium were first analyzed using a chi-squared test. Binary logistic regression was used for a similar analysis with control variables. The significance level was set at *α* = 0.05.

In the binary logistic regression, *survival* is codified as 0 and *failure* as 1. Similarly, *success* is codified as 0 and *not acceptable* as 1. Thus, positive coefficients indicate increased odds of failure or not acceptable, and negative coefficients indicate increased odds of survival or success. Porcelain-veneered CoCr was codified as 1, and porcelain-veneered titanium was codified as 0. If the FDP *length* was 6–12 units, it was codified as 1, and if the FDP length was 3–5 units, it was codified as 0. FDP support type is codified as 1 if the FDP was implant-supported and as 0 if it was tooth-supported. *General tooth wear* was codified as 1 if there was general tooth wear and as 0 if there was no such wear. *Longevity* of the FDP is measured in months.

## Results

### Descriptive data

In total, 63 (55.8%) out of the 113 invited patients were included in the final study group. Table 2 lists the reasons for not participating. The mean age of the included patients was 70.7 years (range 31–86 years), and 39.7% (25 patients) were men and 60.3% were women (38 patients). The mean observation time for all the FDPs was 87.02 months with a minimum of 65 and a maximum of 118 months. For the tooth- and implant-supported FDPs, the mean observation times were 82.09 months (range 65–112 months) and 88.81 months (range 65–118 months), respectively.

**Table 2:** Reasons for non-participation

Deceased	10 patients
Sick	8 patients
Declined	19 patients
Could not find the patient	13 patients

The total number of FDPs, which were delivered by three dental laboratories, was 86. All the metal frameworks were anatomically designed. The number of implant-supported FDPs, all of which were screw-retained, was 53 (61.6%), and the number of tooth-supported FDPs was 33 (38.4%). Sixteen (30.2%) of the implant-supported FDPs were constructed of porcelain-veneered CoCr, and 37 (69.8%) were constructed of porcelain-veneered titanium. The corresponding numbers for the tooth-supported FDPs were 17 (51.5%) and 16 (48.5%), respectively. There were 67 (77.9%) short-span FDPs (3–5 units) and 19 long-span FDPs (6–12 units) (22.1%) (Table 3). The laboratories did not have other detailed information about the material of the FDP framework.

**Table 3:** Frequency distribution of FDP properties

	Number of FDPs
Implant-supported CoCr	16
Implant-supported titanium	37
Tooth-supported CoCr	17
Tooth-supported titanium	16
FDP 6–12 units	19 (7 implant-supported and 12 tooth-supported FDPs)
FDP 3–5 units	67 (46 implant-supported and 21 tooth-supported FDPs)

The majority of the patients were satisfied with the function of their porcelain-veneered CoCr and titanium FDPs, 94.7% and 95.5%, respectively. Similarly, most of the patients were satisfied with the aesthetics of the CoCr and titanium FDPs, 94.7% and 84.1%, respectively. A chi-squared test indicated that the differences were not statistically significant.

The *success* rates of the tooth-supported porcelain-veneered CoCr and titanium FDPs were 64.7% (11 FDPs) and 31.3% (five FDPs), respectively. The corresponding rates for implant-supported FDPs were 62.5% (10 FDPs) and 43.2% (16 FDPs). The *survival* rates of the tooth-supported porcelain veneered CoCr and titanium FDPs were 88.2% (15 FDPs) and 68.8% (11 FDPs), respectively. The corresponding rates for implant-supported FDPs were 62.5% (10 FDPs) and 70.3% (26), respectively. The differences between these success and survival rates for different materials were, however, not statistically significant (Table 4).

**Table 4:** Success and survival rates for FDP materials

	Success rate (number of FDPs)	Survival rate (number of FDPs)
Tooth-supported CoCr	64.7% (11)	88.2% (15)
Tooth-supported titanium	31.3% (5)	68.8% (11)
Implant-supported CoCr	62.5% (10)	62.5% (10)
Implant-supported titanium	43.2% (16)	70.3% (26)

None of the variables had statistically significant effect (Pearson chi-squared) on *success/not acceptable or survival/failure*

None of the implant-supported FDPs were remade, but eight were repaired at a dental laboratory (four porcelain-veneered CoCr and four porcelain-veneered titanium). The number of tooth-supported FDPs that were repaired, by polishing or with composite, at the clinics was three (titanium-porcelain). Metal frameworks did not fracture in either the tooth- or implant-supported FDPs.

### Binary logistical regression analysis

The binary regression analysis, which simultaneously considers the effect of several control variables, indicated that the framework material of CoCr or titanium had no statistically significant effect on the odds of success or survival of tooth- and implant-supported FDPs. Instead, two of the control variables (FDP *length* and *general tooth wear*) had a statistically significant effect on whether the outcome was *satisfactory/success* (R and S) or *not acceptable* (T and V). The odds of the outcome *not acceptable* were 5.27 times higher (*α* = 0.017) when the FDP was long (6–12 units) and 12.9 (*α* = 0.021) when general tooth wear was registered (Table 5). When analyzing the survival of FDPs, FDP *longevity* had a statistically significant effect on the outcome. The odds of failure (V) were 1.07 times higher (*α* = 0.008) for each additional month of longevity (Table 6).

**Table 5:** The effect of FDP material on the odds of FDP being *success/not acceptable*

Variable	Tooth-and implant-supported	Implant-supported	Tooth-supported
FDP Longevity (months)	0.041 (0.021)	0.084 (0.03)*	− 0.049 (0.045)
FDP material (1 = CoCr)	− 0.378 (0.579)	0.059 (0.822)	− 1.471 (1.058)
FDP length (1 = 6–12 units)	1.662 (0.696)*	3.149 (1.587)*	1.186 (0.869)
General wear (1 = general wear)	2.559 (1.110)*	2.779 (1.273)*	20.442 (19,960.069)
FDP support type (1 = implant-supported)	0.064 (0.564)		
Constant	− 6.014 (2.269)	− 10.158 (3.235)	− 15.807 (19,960.00)
Cox & Snell R	0.246	0.338	0.284
Number of FDP: s	86	53	33

Coefficients reported. Positive coefficients indicate increased odds of not acceptable, and negative coefficients indicate increased odds of success. Standard error in parenthesis. Calculations made with SPSS 25

^*^p < 0.05

**Table 6:** The effect of FDP material on the odds of FDP being *survival/failure*

Variable	Tooth-and implant-supported	Implant-supported	Tooth-supported
FDP longevity (months)	0.065 (0.025)*	0.071 (0.031)*	0.067 (0.048)
FDP material (1 = CoCr)	0.914 (0.692)	1.581 (0.967)	0.015 (1.225)
FDP length (1 = 6–12 units)	1.217 (0.674)	2.919 (1.314)*	− 0.073 (0.943)
General wear (1 = general wear)	20.598 (11,465.098)	21.082 (13,965.149)	19.892 (19,885.659)
FDP support type (1 = implant-supported)	0.768 (0.645)		
Constant	− 28.182 (11,465.099)	− 28.901 (13,965.150)	− 26.647 (19,885.660)
Cox & Snell R	0.212	0.301	0.150
Number of FDP:s	86	53	33

Coefficients reported. Positive coefficients indicate increased odds of failure, and negative coefficients indicate increased odds of survival. Standard error in parenthesis. Calculations made with SPSS 25

^*^p < 0.05

However, for the implant-supported FDPs, three of the control variables (*general tooth wear*, FDP *length*, and FDP *longevity*) had a statistically significant effect on whether the outcome was *satisfactory/success* (R and S) or *not acceptable* (T and V). The odds of the outcome *not acceptable* were 16.1 times higher (*α* = 0.029) when general wear was registered, 23.3 (*α* = 0.047) when the FDP was long (6–12 units), and 1.09 (*α* = 0.005) for each additional month of longevity (Table 5).

When analyzing the survival of implant-supported FDPs, two of the control variables (FDP *length* and FDP *longevity)* had a statistically significant effect on the outcome. The odds of failure (V) were 18.5 times higher (α = 0.026) when the FDP was long (6–12 units) and 1.07 (α = 0.020) for each additional month of longevity (Table 6). When analyzing tooth-supported FDPs with the same control variables, there was no statistically significant effect on the odds of success and survival of the porcelain-veneered CoCr or porcelain-veneered titanium FDPs (Tables 5 and 6).
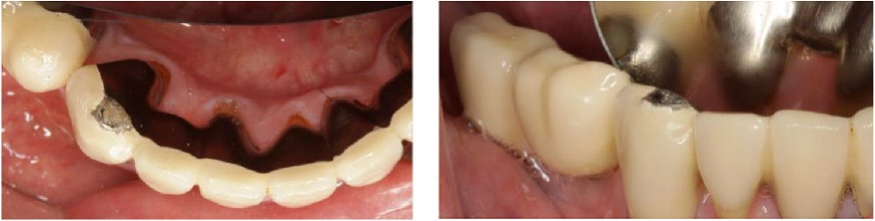


Tooth-supported FDP in porcelain-veneered CoCr with porcelain fracture [43 42 41 31 (32) 33]. The tooth-supported FDP has been in function for over 8 years.
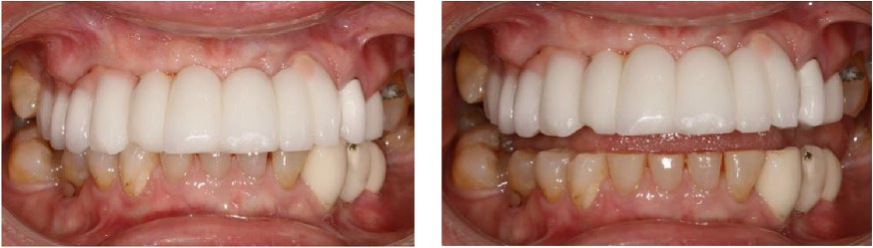


Implant-supported FDP in porcelain-veneered CoCr [(16) 15i 14i (13) 12i (11–22) 23i] with some porcelain fractures. The implant-supported FDP has been in function for over 7 years.

## Discussion

The 63 patients who were part of this retrospective study received their FDPs at one of two prosthodontic specialist clinics. Although the number of FDPs (86) was limited, the follow-up took place 5–9 years after the treatment, which gave an ample opportunity to evaluate both survival and success. This study showed that FDP did not have a statistically significant effect on the odds of success and survival outcomes irrespective of the material used (i.e., porcelain veneered CoCr or porcelain veneered titanium). Thus, the first hypothesis that there are more complications from porcelain-veneered titanium constructions than from porcelain-veneered CoCr constructions was not corroborated. Similarly, whether the FDP was tooth- or implant-supported did not have a statistically significant effect on the odds of success and survival outcomes. Thus, the second hypothesis — i.e., implant-supported FDPs have an increased technical complication rate compared to tooth-supported FDPs — was not supported. No metal frameworks were fractured. Furthermore, the choice of material was not associated with the level of subsequent patient satisfaction.

A limitation of a retrospective design is that it may be difficult to collect all relevant data. These missing data can reduce the power of a study. However, a retrospective design can provide a valuable reflection of the real range and distribution of patients observed in the clinical practice setting [19]. Another limitation of this study is that 55.8% of the invited patients were included in the clinical follow up, which may have affected the results. Moreover, periodontal and radiographic parameters were not recorded.

In the current study, the *success* and *survival* rates of tooth- and implant-supported porcelain-veneered CoCr and titanium FDPs were considerably lower than in previous studies [16, 18, 20, 21]. A possible explanation can be a difference in the tendency to detect minor complications and to report complications in detail between the present and previous studies [22]. Although the differences in Table 4 were not statistically significant, the success rate for both tooth-supported and implant-supported CoCr FPDs was greater than for titanium FDPs. A possible reason for the lack of statistical significance may be the small number of cases in each group.

Since no annual controls were done, this long-term follow-up study did not analyze possible differences in whether porcelain fractures occurred earlier or later during the lifespan of the FDP. It is possible that one of the materials fractured earlier and the fractures increased over time although there was no statistically significant difference after a long-term follow-up. Thus, possible differences between titanium and CoCr may have been evened out.

Previous studies have argued that there are more technical complications (porcelain fractures) with implant-supported FDPs than with the tooth-supported FDPs [18]. One of the reasons for the expected increase in technical complications can be that the natural teeth have periodontal ligaments that provide proprioception while the implants lack this mechanism. The sensory information from periodontal mechanoreceptors is essential for normal control of biting forces [23] to avoid excessive load on the dental restoration, especially on implant-supported FDPs. This reasoning is supported by our results. When analyzing the implant-supported FDPs and tooth-supported FDPs separately, the success of implant-supported FDPs was negatively affected by general wear, whereas tooth-supported FDPs were not. Thus, implant-supported FDPs may be more vulnerable to excessive load.

This study also showed that the risk of fracture of the veneering material was increased with the length of the FDP [21, 22] and among patients with bruxism habits, a finding that agrees with previous studies [21, 24]. It has been reported that the occlusal contact time per night can be about seven times longer among patients with bruxism compared to patients with no bruxism [25]. Therefore, when planning fixed prosthodontics on patients with bruxism, patients should be informed of the higher risks of complications.

In total, 77 of the FDPs in this study were fully veneered (including the occlusal surface), nine were partially veneered occlusally, and three had only metal on the occlusal surface. Because of the small numbers, no statistical comparisons were made. The laboratories did not have detailed information about all the CoCr FDPs—whether they were milled, sintered, or cast. Therefore, the current study did not analyze whether the metal processing method affected the odds of success or survival of FDPs. Previous in vitro research has suggested that, in contrast to titanium, the strength of the porcelain bond to CoCr is higher and not affected by the metal processing technology [26]. The latter findings need to be confirmed in future clinical studies.

The choice of restorative material should be guided by scientific evidence regarding the expected lifespan of the FDP. In practice, however, the choice is often guided by the dentist’s clinical experience and the patient’s wishes. The use of metal ceramics (i.e., gold alloy) has long been a gold standard for rehabilitating teeth [12]. However, the use of monolithic zirconia has increased in both tooth- and implant-supported bridge FDPs and there is a lack of comparative long-term follow-up studies between monolithic zirconia and metal ceramics.

## Conclusion

This study found no statistically significant effect on the odds of success and survival outcomes for any combination of tooth-supported, implant-supported, porcelain-veneered CoCr, or porcelain-veneered titanium FDPs. Thus, the first and second hypotheses were rejected. However, the success of FDPs was negatively affected by greater FDP *length* and *general tooth wear*. Moreover, the survival of FDPs was negatively affected by increased FDP *longevity*.

More studies with larger populations are needed to further evaluate the long-term success and survival of porcelain-veneered CoCr and porcelain-veneered titanium FDPs.
